# Evaluation of the teratogenic potency of bulk zinc oxide and its nanoparticles on embryos of the freshwater snail, *Helisoma duryi*

**DOI:** 10.1038/s41598-024-66008-x

**Published:** 2024-07-10

**Authors:** Manar A. Kandeil, Samia H. Eissa, Hoda K. Salem, Sama S. Hassan

**Affiliations:** Zoology Department, Faculty of Science, Tanta University, Tanta, Egypt

**Keywords:** Teratogenicity, ZnO-BPs, ZnO NPs, Oxidative stress, Protein profile, Biological techniques, Developmental biology, Zoology, Nanoscience and technology

## Abstract

Bulk zinc oxide (ZnO-BPs) and its nanoparticles (ZnO-NPs) are frequently used in various products for humans. *Helisoma duryi* embryos can serve as effective model organisms for studying the toxicity of NPs. This study aimed to compare the teratogenic potency of ZnO-BPs and ZnO NPs in the embryonic stages of *H. duryi* to evaluate the utility of this snail as a bioindicator for ZnO-NPs in the aquatic environment. The mechanisms of teratogenesis were evaluated by determination of the LC_50_, studying the effect of sub-lethal concentrations of both ZnO forms on the embryos, and studying their enzyme activity, oxidative stress, and biochemical analysis. The SDS-PAGE electrophoresis was undertaken to assess the effect of ZnO-BPs and ZnO NPs on protein synthesis. The results revealed that the veliger stage of *H. duryi* is the specific stage for bulk and nano ZnO. ZnO-NPs proved to be more toxic to snails’ embryos than ZnO-BPs. Exposure to ZnO influences specific types of defects in development, which in the case of BPs are far less drastic than those caused by NPs. Thus, the toxicity of ZnO-NPs in embryonic development is due to their unique physicochemical properties. The observed malformations include mainly hydropic malformation, exogastrulation, monophthalmia, shell misshapen, and cell lyses. Almost all tested oxidative biomarkers significantly changed, revealing that ZnONPs display more oxidative stress than ZnO-BPs. Also, the low concentration of ZnO induces many disturbances in the organic substances of veliger larvae, such as a decrease in the total protein and total lipid levels and an increase in the glycogen level. The results indicated that ZnO-BPs increase the number of protein bands. Conversely, ZnO-NPs concealed one band from treated egg masses, which was found in the control group. Embryos of snail are an appropriate model to control freshwater snails. This study demonstrates that *H. duryi* embryos can serve as effective model organisms to study the toxicity of ZnO-NPs.

## Introduction

NPs have various fields of science and technology due to their unique properties and wide-ranging applications^1^. Among these NPs, ZnO-NPs have gathered extensive attention due to their remarkable properties such as UV-blocking ability, high surface area, and antimicrobial properties^2^. However, besides their confirming applications, there is a growing upset regarding their potential toxicity, particularly in biological organisms. Their small size and high reactivity raise concerns about potential adverse effects on the environment^3^. Moreover, their release into aquatic ecosystems through wastewater discharge and runoff poses risks to aquatic organisms^4^.

The toxicity of NPs is critical for several reasons. Firstly, as NPs display increasing use in consumer products, pharmaceuticals, and industrial processes^3^. Secondly, NPs released into the environment can impact ecosystems and wildlife. Studying their toxicity to organisms at various trophic levels helps in predicting and mitigating environmental risks. Thirdly, elucidating the mechanisms of nanoparticle toxicity provides insights into their interactions with biological systems, aiding in the development of safer nanomaterials^5^.

Gastropods, including snails and slugs, are vital components of aquatic and terrestrial ecosystems, playing essential roles in nutrient cycling and food webs^5^. Nanomaterials are toxic to gastropods^6,7^. NPs were used to control the freshwater snail *B. alexandrina* by reducing its fertility^8^. Also, silver nanoparticles NPs have been used as a molluscicide to control land snails^9^ The research on the effects of NP toxicity on gastropods is still an evolving field. Several studies have investigated the effects of NPs on gastropods. Previous studies have demonstrated the susceptibility of gastropods to ZnO-NPs toxicity. ZnO-NPs can enter aquatic environments and accumulate in gastropod tissues through various routes, including dermal exposure, ingestion, and bioaccumulation^10^ Once internalized, they can induce oxidative stress, disrupt cellular functions, and impair vital physiological processes in gastropods^5^. These adverse effects can manifest as reduced survival, growth inhibition, impaired reproduction, and altered behaviour in gastropods, ultimately affecting ecosystem dynamics. Also, reproductive toxicity observed in slugs exposed to titanium dioxide NPs (TiO_2_NPs), indicates potential implications for gastropod populations in contaminated environments^11^.

Moreover, the interactions between NPs and gastropods can be influenced by various factors, including NP size, concentration, surface coating, and environmental conditions. For instance, the surface coating of NPs can affect their bioavailability and toxicity to gastropods^12^. Additionally, the presence of natural organic matter in aquatic environments can modify the interactions between NPs and gastropods, altering their toxicity^13^. The embryonic phase is the most crucial stage of the snail’s life cycle because successful development is essential for the continuation of the snail species and population stability. The present study used ZnO forms (bulk and nano) to evaluate their impacts on the embryonic stages of *H. duryi.*

Gastropods are an appropriate invertebrate model to evaluate the toxic effect of NPs^14–17^. They are an alternate animal and ethically suitable model with biological mechanisms similar to vertebrates^18–20^. Among the freshwater gastropod toxicity tests, the snail embryotoxicity test (SET) is a valuable method for evaluating chemical toxicity^18,21^. The SET methodology for assessing the reproductive impacts of chemical pollutants was established^19,22^. It involves the treatment of the early embryonic developing stage (i.e., blastula stage) to the studied chemical for some days. However, planorbids snail species including *H. duryi,* were used as model organisms in SET^23–26^. The monitored SET endpoints include, juvenile growth rate, and morphological alterations^27–31^. Because these endpoints are highly biologically significant, covering the main embryonic stages that may be combined to affect population level^32^. Furthermore, unlike histological endpoints, they are more susceptible to statistical analysis^33^.

Teratogenicity commonly involves apoptosis, biogenesis, and morphogenesis through mechanisms such as mitotic interference, changes in protein synthesis, deficits in energy precursors, alterations in membrane transport processes, or osmolar imbalance. Teratogens do not necessarily induce the same malformations, nor do phenotypic changes^34^.

As teratogenicity is one of the practical approaches to assessing NP-induced toxicity, the SET is an appropriate test for evaluating the metal toxicity based on NPs and indicating their possible different effects^16,35^.

The main action mechanisms and toxicity of NPs are mainly related to ions produced by the oxidative breakdown of NMs, ROS production, and oxidative stress (i.e., increased lipid peroxidation-LPO changes in the antioxidant defense system). The oxidative stress induced by NPs exposure in snails was studied for many NPs (Ag-NPs, Cd-NPs, CuO-NPs, IONPs, MgO-NPs, TiO_2_-NPs, and ZnO-NPs) and certain snail species, such as *Bellamya aeruginosa*^36–38^, *B. alexandrina*^7^, *L. luteola*^39–41^, and *L. stagnalis*^42^.

Furthermore, NPs can also induce protein carboxylation, DNA damage, and apoptosis^6,37^. Also, they reduce the total protein content and increase the total lipids, alanine aminotransferase (ALT), and aspartate aminotransferase (AST)^7^.

Further research is needed to elucidate the mechanisms of NPs toxicity in gastropods to protect these organisms and their habitats. Understanding how ZnO-NPs affect gastropods and other organisms aids in assessing their ecological implications and instituting measures to reduce their negative effects. Future research should concentrate on uncovering the mechanisms underlying ZnO-NPs toxicity and devising approaches to minimize their environmental impact while optimizing their advantageous uses. The studies about the toxicity of ZnO-NPs on snail embryos are scarce, its toxicity to adult snails has been described for several species, such as *L. luteola*^6^, *B. alexandrina*^7^, and some aquatic and land snails^16^. However, to the best of the writer's knowledge, this is the first study about the toxicity of ZnO forms to snail embryos, indicating that the degree of toxicity is dependent on the developmental stage and that the early stages of snails were more sensitive to ZnO-NPs toxicity.

## Material and methods

### Snail collection

Adult *H. duryi* were collected during the oviposition season, March to October (2022) from many water streams of Mansheyat Ganzour, Tanta, Gharbia, Egypt). The snails were maintained in plastic containers (14 cm depth and 18 cm diameter) with 3 L of dechlorinated tap water (does not contain chlorine or chloramine, pH level = 7.0–7.5) which changed twice a week. They fed mainly on fresh lettuce. They were acclimatized at laboratory room temperature (~ 25 ± 3 °C), with 12 h light/12 h darkness.

### Ethical approval

Tanta University’s Faculty of Science and Institutional Animal Care authorized the experimental protocols and procedures. Experimental procedures followed the International Laboratory Animal Care and Use guidelines. The ethical approval number is IACUC-SCI-TU-0330.

### Experimental chemicals

ZnO-BPs, zinc acetate (Zn (CH_3_COO)_2_) and sodium hydroxide (NaOH) were purchased from El-Gomhouria Co. for Trading Drugs, Chemicals, and Medical Supplies, Tanta, Egypt, and manufactured by Alpha Chemika, Mumbai, India.

Enzymes and MDA were assayed using Automated ELISA System Chemwell 2099 from Gama Trade Company according to the previous methods^43^. The research kits for the application type of ELISA, Kamiya Biomedical Company (catalog no. KT-50849), were used for SOD assay activity and (catalog no. KT-53246) MDA level determination. The research enzyme kits for the application type of ELISA, MyBioSource, (catalog no. KT-53246), were used for the CAT activity assay. The research enzyme kits from Bioassay Laboratory Technology for the application type of ELISA (catalog no. E1172Ra), (catalog no. E1172Ra), and (catalog no. 703002) were used for GPx, GST, and GSH activity assays, respectively.

#### Synthesis and characterization of ZnO-NPs

ZnO-NPs were prepared in our laboratory using the low-temperature aqueous method. 1.8 g of zinc acetate (Zn (CH_3_COO)_2_) was dissolved in 90 ml of distilled water and vigorously stirred for 75 min using an ultrasonic processor. 9 ml of 3 M NaOH was added gradually to the zinc acetate solution after 15 min of ultrasonication then filtrate to obtain white precipitate that washed with distilled water. It was dried overnight at 60 °C in an oven and then was manually ground with a mortar and pestle to obtain a fine powder.

The morphological characters and particle size of ZnO-BPs and synthesized ZnO-NPs were investigated and visualized in our previous study^44^ using scanning electron microscopy (SEM; JEOL JSM-5300) transmission electron microscopy (TEM; JEOL JEM-2100). The particles were examined at different resolutions and magnification powers in a random field of view. X-ray Powder Diffraction (XRD Shimadzu 6000) analyses were performed previously at Nano Science and Technology Institute, Kafrelsheikh University, Egypt^44^.

### Egg masses and embryos

*H. duryi *lays daily its egg masses, either on polyethylene sheets or on the surface wall of the aquarium. The egg masses were collected with a clean spatula and kept in Petri dishes.

### Developmental stages and experimental design

#### Chronic effect of sub-lethal concentrations of bulk and nano ZnO on the fertilized egg

The experimental samples were composed of five freshly laid egg masses, each containing about 20 ± 5 viable embryos. Egg masses were placed in glass Petri dishes and washed with clean water before the commencement of the experiment. The egg masses were randomly assigned to two groups: Group I was the control, and Group II was under the chronic effect of ZnO-BPs and ZnO-NPs from fertilized egg till the end of development. The mortality and malformation percentages of each embryonic stage (fertilized egg & cleavage, blastula, gastrula, trochophore larva, and veliger larva) were determined according to the LC_90_, LC_50,_ and LC_10_ investigation using probit analysis^45^. The lethal concentrations of embryos were investigated by exposing the egg masses of *H. duryi* to serial concentrations of ZnO-BPs and ZnO-NPs (300, 600, and 900 µM) and (6, 10, 30, 60 µM), respectively. The test suspensions were diluted from stocks with dechlorinated water in Petri dishes which were filled with 50 ml of each dilution. The percentage of mortality was calculated as the number of dead embryos divided by the total number of developing embryos × 100^19^. Five freshly laid egg masses containing 20–30 eggs were incubated at 25 ± 2 °C in each Petri dish for either control or exposed groups. Three replicates were established for control and each concentration. The mortality was recorded at the end of each embryonic stage.

The teratogenicity assay was done at each embryonic stage (cleavage stage, blastula, gastrula, trochophore, and veliger stages). The sub-lethal concentrations for ZnO-BPs and ZnO-NPs were under LC_10_ (3, 6, 10 µM) and (0.1, 0.3, 0.8 µM), respectively. Three replicates were used for each group. Egg viability criteria depend on the motility of the embryo within the egg. The effects on different embryonic stages were examined and photographed using a camera linked to a dissecting microscope to detect the embryonic abnormalities. The most affected developmental stage (specific stage) and the most effective concentration yield were detected.

#### Effect of sub-lethal concentrations of bulk and nano ZnO *on major* developmental stages

At the same experimental conditions and sub-lethal concentrations, the egg masses were randomly assigned to two main groups; Group I was the control. Group II Includes the effect of ZnO-BPs and ZnO-NPs on the cleavage stage (from fertilized egg till the end of cleavages), blastulation and gastrulation stage (from blastula till the end of gastrulation) and larval stage (from trochophore larva till hatching). Larvae with a detached foot were tested by stimulating the foot with the bristles of a fine brush. Embryos with no heartbeat, rotational or foot movements, and disaggregated embryos were considered dead. These observations are according to the snail embryotoxicity test (SET)^46,47^.

#### The effect of the time factor on the specific stage exposed to both ZnO forms

According to experiments 1 and 2, egg masses containing the determined specific stage were exposed to the most effective concentrations of ZnO-BPs and ZnO-NPs to show the effect of time and compare the effectiveness of both ZnO forms.

### Biochemical studies

Biochemical studies were conducted in the Central Lab of the Faculty of Science at Tanta University. The total protein, lipid, glycogen, protein bands, and oxidative stress enzymes were determined as biochemical parameters of control, and malformed veliger larvae were exposed to 10 µM ZnO-BPs and 0.4 µM ZnO-NPs. For each group, 25 ± 5 egg masses were frozen at − 20 °C until the analysis time.

#### Enzymes activity of egg masses and oxidative stress analysis

##### Preparation of specimens

The samples were crushed inside a glass homogenizer in ice-cold phosphate buffer (50 mM phosphate pH 7.4), 10% (W/V) using Omni international homogenizer (USA) at 22,000 rpm for 20 s each with 10 s intervals. The homogenate was centrifuged at 2000 Xg in the cooling centrifuge (Hettich, Germany) at four °C for 15 min, the obtained supernatant was saved. The supernatant was freeze-thawed twice to disrupt it completely (Salach Jr, 1978). Then, the supernatant was centrifuged again at 6000 Xg at four °C for 15 min, and the yielded supernatant containing the cytosolic and mitochondrial enzymes was saved for rapid enzyme assay. The supernatants were used for the measurement of catalase (CAT), glutathione S-transferase (GST), glutathione peroxidase (GPx), Superoxide dismutase (SOD) activities, malondialdehyde (MDA) levels, and reduced glutathione (GSH) content.

##### SOD and CAT assessment

The supernatants were used for the measurement of SOD and CAT according to the previous method^48,49^.

The results of SOD activity were expressed as the number of mg wet-weight egg masses that inhibit 50% of reactive oxygen species (ROS) production.

The results of CAT activity were expressed as µM/min/g wet-weight egg masses. Both kits used for either SOD or CAT apply a linked immunosorbent assay application technique (quantitative sandwich immunoassay)^50,51^. The microtiter plate in these kits has been pre-coated with a polyclonal antibody specific for SOD and CAT, respectively.

##### GPx, GST, and GSH assay protocols

40 µl of the sample and 10 µl of antibody (anti-GPx-ab, anti-GST-ab, or anti-GSH-ab) were placed, then 50 µl streptavidin HRP were added to each well. For 1 h, the mixture was incubated at 37 °C. The wash buffer was used to wash the plate five times. 50 µl of substrates (A and B) were placed and incubated at 37 °C for 10 min. 50 µl of stop solution was added. The wavelength was determined at 450 nm.

The result of GPx activity was mentioned as µM/min/g wet weight of egg masses and applied to the competitive enzyme immunoassay technique^52,53^ using a polyclonal anti-GPx antibody with a GPx-HRP conjugate.

The results of GST activity were expressed as optical density per gram of protein per minute.

The amount of reduced GSH present in the egg mass samples in terms of g/g of wet sample.

##### Malondialdehyde (MDA)

Lipid peroxidation (LPO) products were determined as the quantity of malondialdehyde (MDA) produced and applied using the quantitative sandwich enzyme immunoassay technique^54^. A monoclonal antibody which is specific for MDA has been used to pre-coat the microtiter plate has been. The results are expressed as nM/mg of wet egg masses.

### Biochemical assessment

Biochemical parameters were detected in the supernatant of the tissue homogenate. The total glycogen in embryos was determined according to the previous study^55^ with some modifications. A standard curve was constructed using standard samples prepared from starch dissolved in distilled water, with different concentrations (2%, 4%, 6%, …, 10%). The amount of glycogen was determined from the prepared standard curve.

The total lipid in the egg masses of the snails was determined using a commercial kit purchased from Biodiagnostic (Egypt). This method depends on the reaction of lipids with vanillin in a medium of sulphuric and phosphoric acids to form a pink-colored complex^56^. Phosphate-buffered saline (PBS, pH 7.4) has been used to homogenize samples. Then, the absorbance was measured at wavelengths of 530–560 nm. Olive oil standards were used for standard curves ranging between 5 and 40 mg/dl.

Total protein in the egg masses was determined according to Tietz^57^, using a commercial kit (Biomed Diagnostics, 30,175 Hannover, Germany). Standard samples were prepared from Bovine serum albumin dissolved in distilled water. Samples were measured against a blank reference containing distilled water.

#### Electrophoretic studies of protein bands

SDS-PAGE (Sodium dodecyl sulfate–polyacrylamide gel electrophoresis) was prepared using the method of Laemmli^58^. This technique was used to separate proteins depending on their molecular weights after staining with Coomassie stain. The given lane’s percentage of shared bands is compared to those in other lanes of the same gel. The electrophoretic studies of protein bands were carried out at the Scientific Research Center and Measurements (SRCM), Tanta University.

#### Preparation of the gels

The stacking gel was composed of 0.5 ml 30% acrylamide mixture, 0.38 ml of 0.5 M Tris-buffer (pH 6.8), 0.03 ml 10% ammonium persulfate, 0.003 ml TEMED (*N*,*N*,*N*′,*N*′-tetramethylene diamine), 0.03 ml 10% SDS (Sodium dodecyl sulfate) and 2.1 ml H_2_O. The separation gel was composed of 5 ml of 30% acrylamide mixture, 3.8 ml of 1.5 M Tris-buffer (pH 8.8), 0.15 ml 10% ammonium persulfate, 0.006 ml TEMED, 0.15 ml 10% SDS 10% (5 g in 50 ml of distilled water), and 5.9 ml of H_2_O.

Electrophoresis was carried out after adding the running buffer at a constant voltage (100 V) until the marker reached the gel's bottom. The separation took 3–5 h, and then the gel was removed and placed in the staining solution overnight.

#### Staining of the gels

Using the Coomassie staining technique, the gels were stained in a solution composed of 50 ml acetic acid, 250 ml methanol, and 62.2 ml of 1% Coomassie brilliant blue (R250) and made up to 500 ml of distilled water.

#### Destaining of the gels

The gels were destained to remove the background stain by shaking in 50 ml of destaining solution (10% acetic acid + 40% methanol). The gels were then analyzed and photographed. Gel documentation system (Geldoc-it, UVP, England) was applied for data analysis using TotalLab analysis software Ver.1.0.1.

#### Microphotography and digital image processing

Living embryos of different developmental stages inside egg masses were observed and photographed using a camera mounted on a conventional dissecting light microscope. Digital images were analyzed, and the size of scale bars was determined using ImageJ software.

### Statistical analysis

All statistical data were analyzed with ANOVA test (one-way analysis of variance) to determine the statistically difference between the groups. All statistics were performed using SPSS software. The data were presented as mean ± SD of three independent groups, each performed in triplicate. Differences between the treated samples and controls were considered statistically significant for *p*-values < 0.05.

## Results

### ZnO-BPs and ZnO-NPs characterization

**Figure 1:**
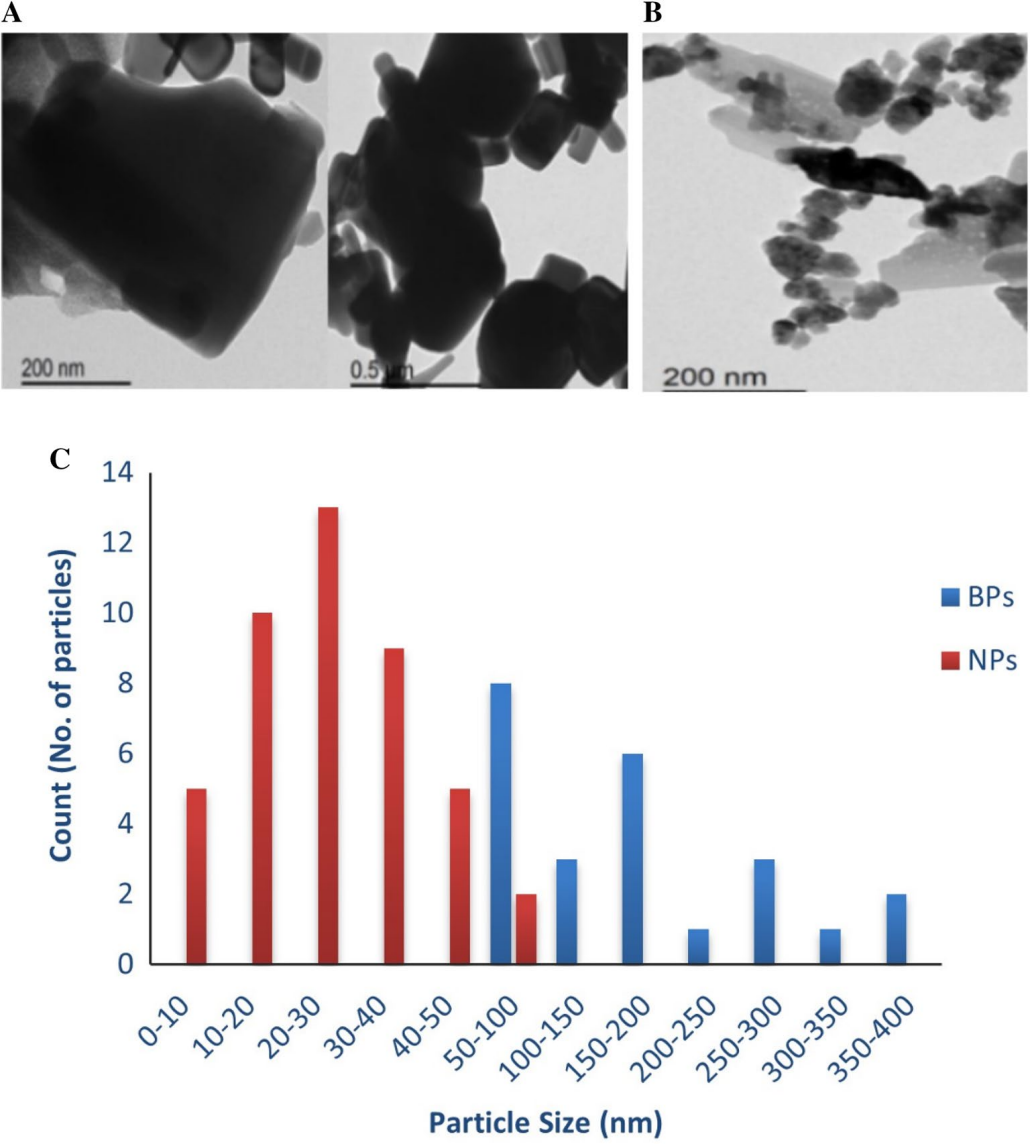
TEM micrograph of ZnO-BPs (**A**) and ZnO-NPs (**B**); Histogram of particle size distribution from TEM photograph of ZnO-BPs and ZnO-NPs (**C**).

The TEMmicrographs of ZnO-BPs and ZnO-NPs that are shown in Fig. 1A,B confirmed that ZnO-BPs were more irregular with a rectangle or square-like shape, whereas ZnO-NPs were hexagonal (spherical and rod-shaped), with some particles possessing edges. The average particle sizes distribution histograms of ZnO-BPs and ZnO-NPs (Fig. 1C) were 165.36 ± 54.04 and 21.94 ± 5.19 nm, respectively which calculated from the TEM micrographs by the ImageJ software.

### LC_10_, LC_50_, and LC_90_, determination of ZnO-BPs and ZnO-NPs in different developmental stages of *H. duryi*

The lysis of embryonic cells or the absence of movement of the embryos was considered the parameter for calculating the mortality percentage of developmental stages. The LC_10_, LC_50_, and LC_90_ values of ZnO-BPs and ZnO NPs against different developmental stages (cleavages, blastula, gastrula, trochophore, and veliger stages) of *H. duryi* have been summarized in Table 1, 2. The mortality percent values were determined after exposure of all developmental stages to a series of ZnO-BPs and ZnO-NPs concentrations (300–1200 µM) and (6–60 µM), respectively. The exposure time is from the beginning of each stage till its end.

**Table 1:** Lethal and sub-lethal concentrations along with 95% confidence limits of ZnO-BPs on different developmental stages of *H. duryi* after exposure to some concentrations. The recorded time is from the beginning of each embryonic stage.

Developmental stages	Exposure time (h)	ZnO-BPs (µM)		
		LC10	LC_50_	LC_90_
Fertilized egg & cleavage stages	7	79 ± 10 (60–90)	82 ± 11 (70–90	90 ± 20 (70–100)
Blastula stage	12	65 ± 20 (40–80)	68 ± 14 (54–80)	72 ± 16 (50–90)
Gastrula stage	12	149 ± 30 (120–180)	152 ± 30 (120–190)	157 ± 30 (120–190)
Trochophore	12	43 ± 10 (30–50)	45 ± 10 (30–60)	48 ± 14 (30–60)
Veliger larva	30	15 ± 3 (16–18)	16 ± 4 (11–20)	20 ± 2 (18–22)

**Table 2:** Lethal and sub-lethal concentrations along with 95% confidence limits of ZnO-NPs on different developmental stages of *H. duryi* after exposure to some concentrations.

Developmental stages	Exposure Time (h)	ZnO-NPs (µM)		
		LC_10_	LC_50_	LC_90_
Fertilized egg & cleavage stages	7	5.7 ± 1 (4.6–6.8)	5.8 ± 0.2 (5.6–6)	5.9 ± 1 (4.8–7)
Blastula stage	12	5.9 ± 3 (3.6–8.2)	6 ± 3 (2.6–0.9)	6.3 ± 0.6 (5.6–7)
Gastrula stage	12	1 ± 0.1 (0.9–1.1)	1.2 ± 0.2 (0.1–1.4)	1.2 ± 0.3 (0.9–1.5)
Trochophore	12	5 ± 0.2 (4.8–5.2)	5.2 ± 0.5 (4.6–5.7)	5.3 ± 0.6 (4.6–6)
Veliger larva	30	2 ± 0.3 (1.7–2.3)	2.2 ± 0.4 (1.8–3	2.3 ± 0.2 (2.1–2.3)

The recorded time is from the beginning of each embryonic stage.

### The mortality rate of developmental stages exposed to different concentrations of ZnO

The mortality rate induced by ZnO-BPs and ZnO-NPs is concentration and time-dependent. All developmental stages showed the highest mortality rate after the exposure time recorded from the beginning of each developmental stage and at the highest exposed concentrations of ZnO-BPs and ZnO-NPs (900 and 1200 µM) and (30 and 60 µM), respectively. The mortality rate for ZnO-NPs at high concentrations was higher in many cases than that of bulk, as shown in Table 3. There was no effect at the beginning of each stage, especially after ZnO-BPs exposure. After some time, the influence became evident and increased gradually till the end of each developmental stage. It was expected that ZnO-BPs would penetrate slowly due to their large size and the high resistance of the capsular membrane. In contrast, the nano-form was more effective, which may result from its minimal size.

**Table 3:** The mortality rate of *H. duryi* developmental stages exposed to some concentration of ZnO-BPs and ZnO-NPs.

Developmental stages	Time of exposure (h)	Mortality percentage							
		ZnO-BPs (µM)				ZnO-NPs (µM)			
		300	600	900	1200	6	10	30	60
Fertilized egg & cleavage stages	1	0	0	0	0	0	0	0	0
	2	0	0	0	0	0	2.5 ± 0.4	4.9 ± 0.8	8 ± 1
	6	49.8 ± 3.3	67.9 ± 0.5	72.4 ± 2.2	100	0	66.7 ± 4	100	100
Blastula stage	1	0	0	0	0	0	0	0	0
	6	0	12 ± 2	21.5 ± 3.5	35.1 ± 2.8	6.5 ± 1.6	8.2 ± 0.7	19.5 ± 2	25.4 ± 2
	12	43.8 ± 2.5	57.1 ± 3	65.6 ± 4.6	79.9 ± 6	11.1 ± 0.7	20.7 ± 3.6	80.13 ± 7	96.7 ± 3
Gastrula stage	1	0	0	0	0	0	0	0	0
	6	3.7 ± 1.4	11.4 ± 3	35.1 ± 2.8	55.5 ± 3.5	0	17.9 ± 1.6	49.8 ± 3	78.4 ± 5
	12	31.6 ± 2	92.3 ± 5	100	100	44.1 ± 4.5	64.8 ± 6	98.5 ± 8	100
Trochophore	1	0	0	0	0	0	0	0	0
	6	0	25.5	43.2	60.4	7.21 ± 0.9	46.2 ± 6.5	52.9 ± 3	78.4 ± 8
	12	58.9 ± 4	61.8 ± 7	82.9 ± 5.8	94.1 ± 5	0.00	84.6 ± 4	100	100
Veliger larva	1	0	0	0	0	0	0	0	0
	6	0	0	9.5 ± 3	19.1 ± 2	3.2 ± 1	3.9 ± 0.5	7.6 ± 2	15.4 ± 4
	12	4.7 ± 0.2	9.2 ± 1.4	14 ± 2	37.2 ± 6	8.2 ± 0.4	12.5 ± 0.6	19.1 ± 5.6	34.9 ± 6
	30	67.4 ± 3	75 ± 6	78.64 ± 8	100	38.1 ± 3.8	46.8 ± 8	84.6 ± 4	100

The time recorded for each stage is from the beginning of it.

### Chronic effect of ZnO from the fertilized egg till the end of development

**Figure 2:**
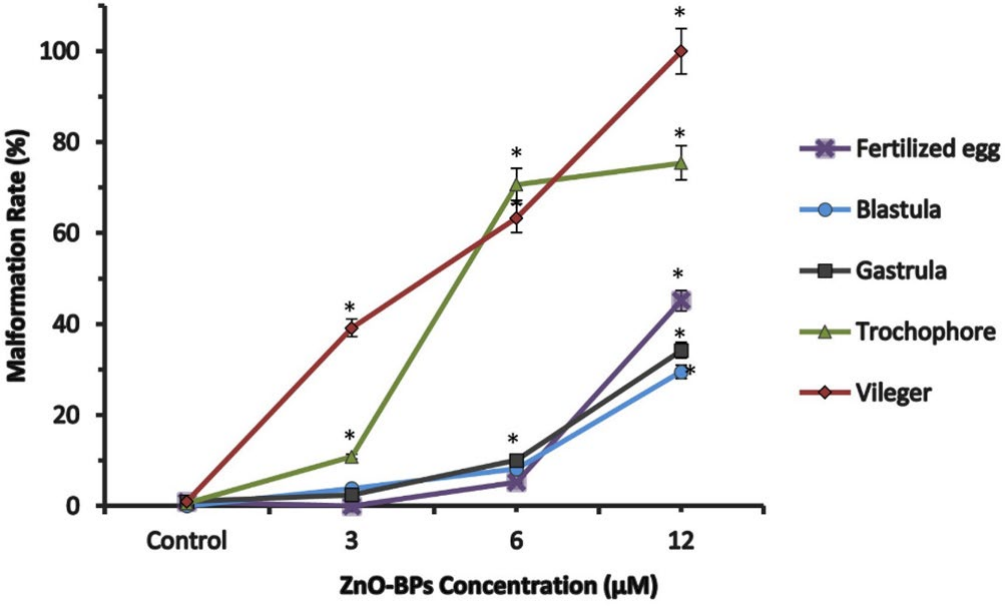
Percentage of malformed embryos (fertilized egg, cleavages stage, blastula, gastrula, trochophore, and veliger) after eight days of exposure with various concentrations of ZnO-BPs (exposure from the fertilized egg). The data are expressed as mean + SD of n = 3 independent experiments performed in (triplicate), Statistical analysis: One-way ANOVA, *p < 0.05 versus control.

**Figure 3:**
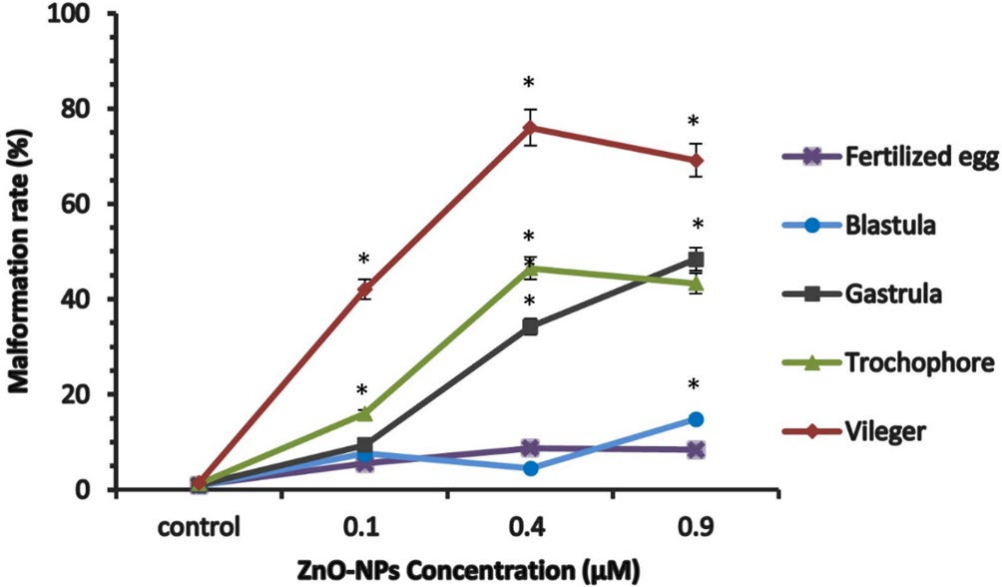
Percentage of malformed embryos (fertilized egg, cleavages stage, blastula, gastrula, trochophore, and veliger) after eight days of exposure with various concentrations of ZnO-NPs (exposure from the fertilized egg). The data are expressed as mean + SD of n = 3 independent experiments performed in (triplicate), Statistical analysis: One-way ANOVA, *p < 0.05 versus control.

The effect of sub-lethal concentrations of ZnO-BPs and ZnO-NPs (3, 6, 10 µM) and (0.1. 0.4, 0.9 µM) respectively, on the developmental stages of *H. duryi*, was shown in Figs. 2 and 3.

The fertilized egg and early cleavages gave lower malformation percentages than the trochophore and veliger stages. Veliger larva was the most malformed stage.

The malformation percentage increased with the increase in the concentration of ZnO-BPs. As shown in Table 4, the malformation percentage depended on the concentration of ZnO-BPs and the exposure time. Figure 2 showed that 10 µM ZnO-BPs was the most effective concentration at which all developmental stages showed the highest percentage of malformations. The development was arrested at the veliger stage at this concentration, and its lyses began after 6–7 days of egg-laying.

**Table 4:** Relation between the embryonic stage exposed to ZnO (bulk and nano forms) and the percentages of abnormal embryos after 8 days of treatment (The total period of the development).

Developmental stage	Time after egg laying (h)	Percentage of abnormal embryos after 8 days					
		ZnO-BPs (µM)			ZnO-NPs (µM)		
		3	6	10	0.1	0.4	0.9
Fertilized egg	2	0	5.23 ± 0.4	45.11 ± 3	5.56 ± 0.5	8.76 ± 0.9	8.43 ± 0.6
Blastula	12	3.86 ± 1	8.15 ± 1.3	29.5 ± 2	7.57 ± 0.8	4.54 ± 0.4	14.82 ± 0.5
Gastrula	24	2.46 ± 0.5	10.06 ± 3	34.21 ± 6	9.37 ± 0.6	34.24 ± 2	48.34 ± 4
Trochophore	36	10.84 ± 2	70.67 ± 7	75.4 ± 4.6	15.98 ± 2	26.47 ± 4	53.34 ± 6
Veliger	66	39.12 ± 4	63.29 ± 5	99.96 ± 1	32.03 ± 3	45.76 ± 5	75.99 ± 5

Although the sensitivity of fertilized eggs to low concentrations of ZnO-NPs (0.1, 0.4, 0.9 µM) was high, cleavage was established, and embryonic development proceeded to the late larval stages. The percentage of abnormal embryos depended on the concentration of ZnO-NPs and the developmental stage, as shown in Fig. 3 and Table 4. At0.9 µM, the veliger stage could not complete its development due to the greater efficacy of nano-zinc oxide than that of bulk particles.

### Effect of ZnO forms on three major stages of development (cleavage, blastulation and gastrulation, and larval)

**Figure 4:**
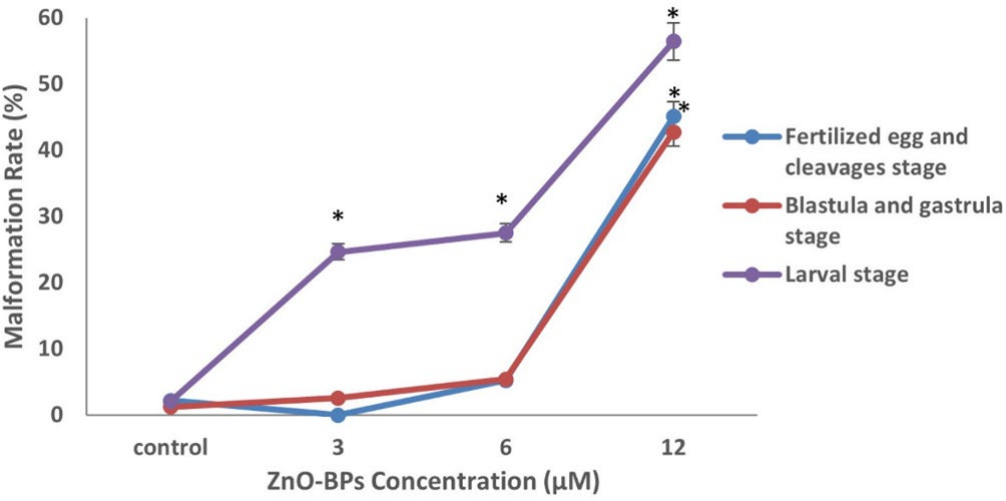
Percentage of malformed embryos (Fertilized egg and cleavage, blastulation and gastrulation, and larval stages) after exposure to various concentrations of ZnO-BPs (exposure from the beginning of each stage). The data are expressed as mean + SD of n = 3 independent experiments performed in (triplicate), Statistical analysis: One-way ANOVA *p < 0.05 versus control.

**Figure 5:**
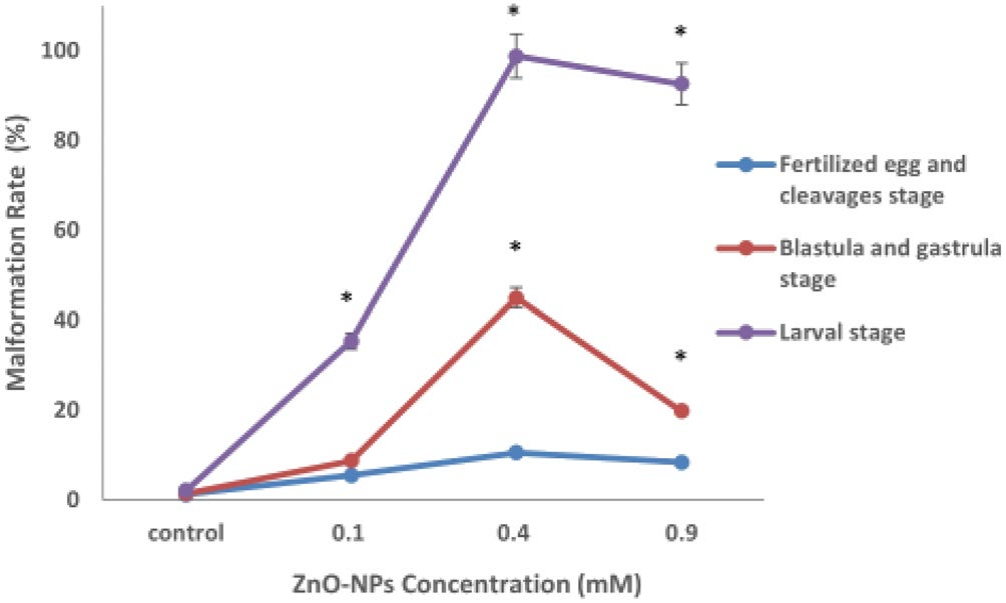
Percentage of malformed embryos (Fertilized egg and cleavage, blastulation and gastrulation, and larval stages) after exposure to various concentrations of ZnO-NPs (exposure from the beginning of each stage).The data are expressed as mean + SD of n = 3 independent experiments performed in (triplicate), Statistical analysis: One-way ANOVA *p < 0.05 versus control.

The effect of sub-lethal concentrations of ZnO-BPs from the beginning of each developmental stage is shown in Fig. 4. The exposure of fertilized eggs and blastulae stages to the lowest ZnO-BPs tested concentration (3 µM) scarcely produced abnormal embryos. On the other hand, the higher concentration (10 µM) of ZnO-BPs can induce a high percentage of abnormalities, especially at the larval stage. 75–100% malformation percentage was obtained during embryos' exposure to ZnO-BPs at the veliger stage. So, this stage can be considered a specific stage at which embryogenesis is highly sensitive to ZnO-BPs. The most effective ZnO-NPs concentration at which most stages displayed a higher percentage of malformations was 0.4 µM (Fig. 5). The malformation percentage that exceeds 75% was obtained during embryos’ exposure to higher sub-lethal concentrations (0.4 and 0.9 µM) of ZnONPs at the veliger stage. Hence, as in ZnO-BPs, this stage can be considered a specific stage at which embryogenesis is highly sensitive to ZnO-NPs. There were almost no mortalities during cleavage, blastulation, and gastrulation stages after exposure to ZnO-BPs, compared to the mortality rate after ZnO-NPs exposure (Table 5).

**Table 5:** Relation between the major developmental stages exposed to ZnO (bulk and nano forms) and the mortality percentage after each stage (cleavage, blastulation and gastrulation, and larvae stages).

Developmental stage	Time of exposure	Percentage of dead embryos after each stage					
		ZnO-BPs (µM)			ZnO-NPs (µM)		
		3	6	10	0.1	0.4	0.9
Cleavages	2 h	0	0	0	0	0	0
	6 h	0	0	18 ± 2	2.41 ± 1	3 ± 0.7	9.85 ± 2
Blastulation and gastrulation	12 h	0	3 ± 0.6	4.65 ± 0.5	0	0	0
	24 h	2.58 ± 0.6	12.74 ± 3	11.8 ± 2.9	2.56 ± 1.5	4.23 ± 0.7	14.83 ± 3
Larval stage	2 d	1 ± 0.2	3.88 ± 1	6.73 ± 3	3.21 ± 0.4	4.12 ± 0.5	9.57 ± 2
	4 d	4.56 ± 0.5	15.1 ± 2	18.25 ± 1	9.58 ± 2	13.94 ± 1.7	20.77 ± 3

**Figure 6:**
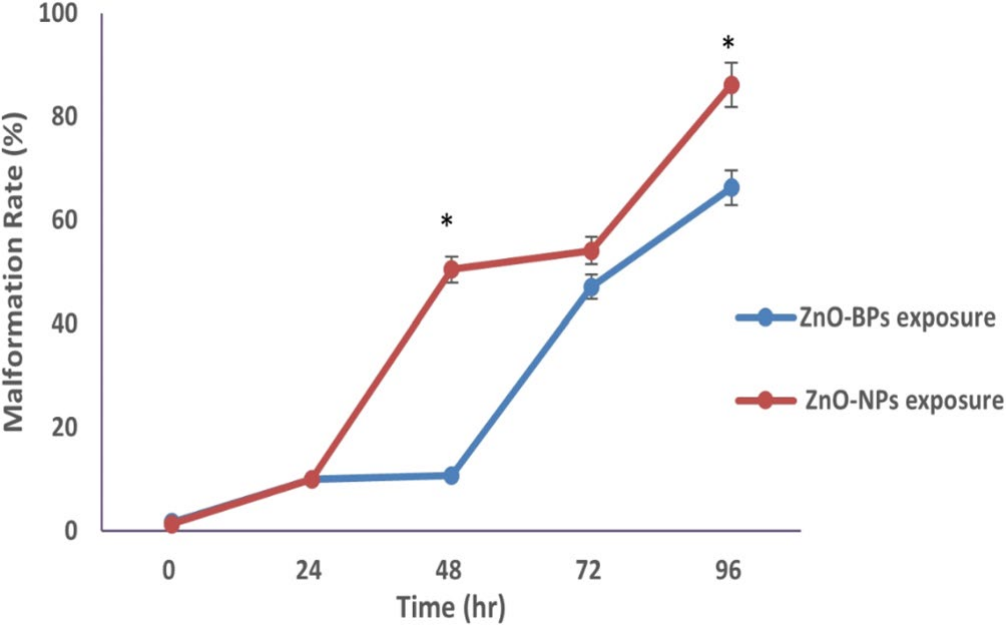
Comparison between malformation rate of veliger stage. *p < 0.05 versus control.

**Figure 7:**
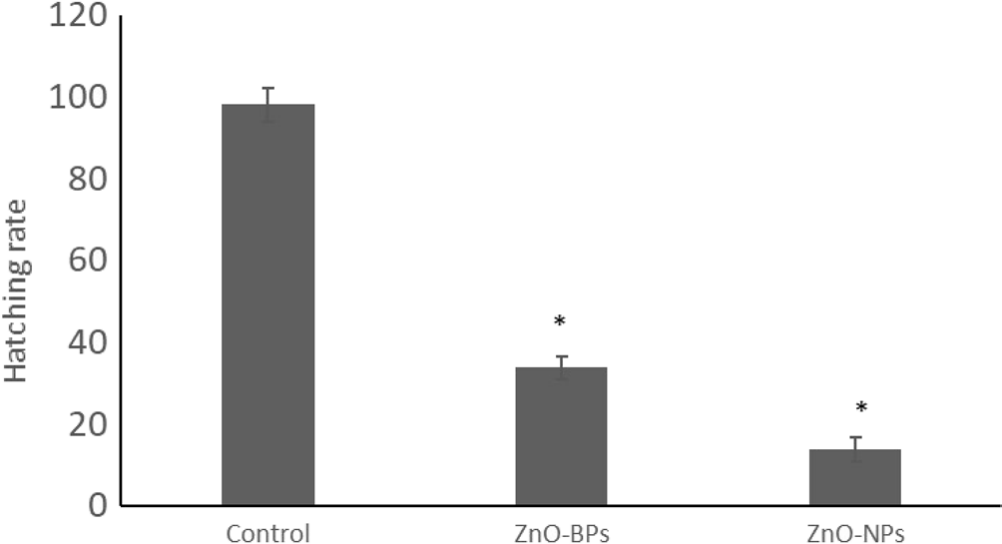
Comparison between hatching rate of veliger stage treated with both ZnO-BPs (10 µM) and ZnO-NPs (0.4 µM). *p < 0.05 versus control.

Conversely, the larval stage exhibited a higher mortality percentage than other stages. Thus, the mortality rate for both ZnO forms depends on concentration, time, and exposed stage. Time of exposure is another factor that directly affects the malformation percentage in veligers exposed to both bulk and nano ZnO. Figure 6 indicated the higher effectiveness of ZnO-NPs than that of ZnO-BPs, as the exposed veligers showed higher malformation rates during each time interval. The hatching rate of exposed veligers was expected to decrease significantly compared to the control group, especially for ZnO-NPs (Fig. 7). In addition, the hatching snails were divided into normal and malformed.

### Morphological alterations (Malformations)

**Figure 8:**
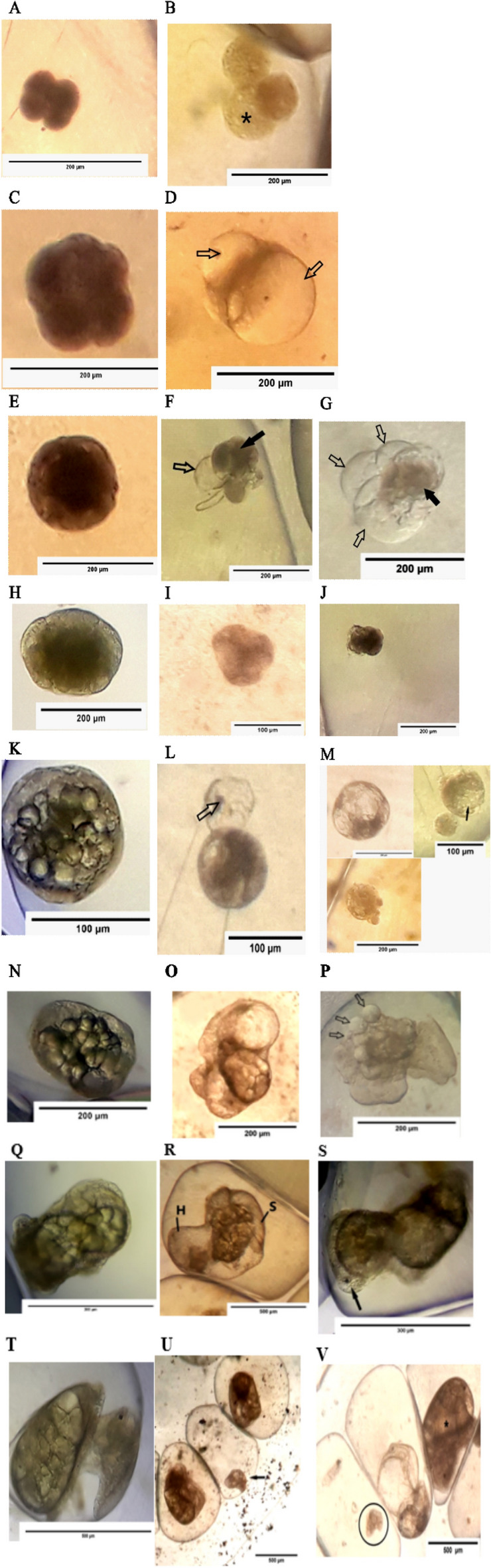
Photographs of abnormal embryonic stages treated with ZnO-BPs and ZnO-NPs. All photos were taken by a digital camera linked to a light dissecting microscope. (**A**) Normal 4-cell stage. Scale bar: 200 µm. (**B**) Abnormal 4-cell stage exposed to 0.4 µM ZnO-NPs showing the disrupted blastomeres. The star indicates the unknown vesicle. Scale bar: 200 µm. (**C**) normal morula. Scale bar: 200 µm. (**D**) Abnormal morula exposed to ZnO-NPs. The arrows indicate large blebs. Scale bar: 200 µm. **E:** Normal blastula. Scale bar: 200 µm. (**F**) Abnormal blastula exposed to ZnO-BPs. The arrow indicates the bleb. The black arrow indicates the compact cells and blastocoel. Scale bar: 200 µm. (**G**) Abnormal blastula exposed to ZnO-NPs. The arrows indicate large blebs. The black arrow indicates the compact cells and blastocoel. Scale bar: 200 µm. (**H**) Normal gastrula. Scale bar: 200 µm (**I**) Abnormal gastrula exposed to ZnO-BPs. Scale bar: 100 µm. (**J**) Abnormal gastrula exposed to ZnO-NPs. Scale bar: 200 µm K**:** Normal trochophore. Scale bar: 100 µm (**L**) Abnormal trochophore exposed to ZnO-BPs. The arrow indicates the bleb. Scale bar: 100 µm. (**M)** Abnormal trochophore exposed to ZnO-NPs showing hydropic malformation. The black arrow indicates the vacuolation. Scale bar: 100, 200 µm (**N**) Normal veliger. Scale bar: 200 µm (**O**) Abnormal veliger exposed to ZnO-BPs with hydropic and strikingly nonspecific malformations. Scale bar: 200 µm (**P**) Abnormal veliger exposed to ZnO-NPs. The arrows indicate the blebs. Scale bar: 200 µm (**Q**) Normal late veliger. Scale bar: 500 µm. **R:** Abnormal late veliger exposed to ZnO-BPs showing cephalic hydropic malformation and shell misshapen. Head (H), Shell (S). Scale bar: 500 µm (**S**) Abnormal late veliger exposed to ZnO-NPs. The black arrow indicates monophthalmia at the right. Scale bar: 300 µm. (**T**) Normal larva. Scale bar: 500 µm (**U**) Abnormal larvae exposed to ZnO-BPs showing retarded growth (black arrow) and normally developed embryos. Scale bar: 500 µm (**V**) Abnormal larvae exposed to ZnO-NPs showing hydropic malformation, shell misshapen, and growth retardation. The star shows the normal pre-hatching juvenile. The black circle indicates retarded growth veliger. Scale bar: 500 µm.

Morphological alterations in *H. duryi* embryos are shown in Fig. 8. When egg masses were exposed to distinct sub-lethal concentrations of ZnO-NPs and ZnO-BPs, the embryos displayed characteristic alterations in the normal development pattern at different stages. The degree of malformation varied from slight to severe according to the exposure time and concentration of ZnO.

Compared with the control 4-cell stage, the treated 4-cell stage with ZnO-NPs showed some abnormalities, such as asymmetric cleavages, blastomere disintegration, and decreasing blastomeric cohesion. Sometimes, the disrupted blastomere appeared as an unknown vesicle with many small vacuoles (Fig. 8A,B). As the development proceeds, the morula stage showed strikingly dysmorphic anomalies and presented ideal hydropic malformation as it was greatly swollen and formed large blebs that not present in the control (Fig. 8C,D). These embryos rarely hatched or frequently died after hatching.

Figure 8E–G showed control and treated blastulae showed degeneration of ectodermal cells with compact blastocoels7. Some large blebs protruded around the margin of the blastula and were never present on control ones. In comparison to control, the malformed gastrulae were marked with compacted embryonic cells without any tissue organization and so much slightly small evagination of the gut (exogastrula) (Fig. 8H–J). Progressively, the cells of gastrula were dissociated and degenerated, leading to the death of this stage.

The control and treated larval stages are shown in Fig. 7K–M). The exposed larvae to ZnO-BPs were characterized by protrusion of large external bleb (Fig. 8L). Thelarval cells treated with ZnO-NPs degenerated and lysi and could not develop further (Fig. 8M). The dysmorphic developed trochophore larva showed a highly swollen hydropic shape and gradual vacuolation and dissociation displayed on its cells (Fig. 8M).

Control and treated veliger and pre-hatching juveniles are shown in Fig. 8N–V. The treated veliger larvae with ZnO-BPs showed hydropic and strikingly nonspecific malformations (Fig. 8O). The treated veliger larvae with ZnO-NPs showed a considerable degree of hydropic malformation as they were partly or totally swollen. many small to medium-sized blebs were formed around the margin of the larvae (Fig. 8P), which means losing one eye. Also, shell misshapen occurred in some pre-hatching juveniles (Fig. 8R). Some larvae showed head malformation as monophthalmia (Fig. 8S). Growth retardation is another type of malformation in veligers and pre-hatching ones (Fig. 8U,V). Mostly, these larvae were disorganized and underwent lysis and death after their cells were dissociated and degenerated.

### Biochemical effects of bulk and nano ZnO on veliger larvae (specific stage)

#### Oxidative stress and embryonic damage caused by bulk and nano ZnO

**Figure 9:**
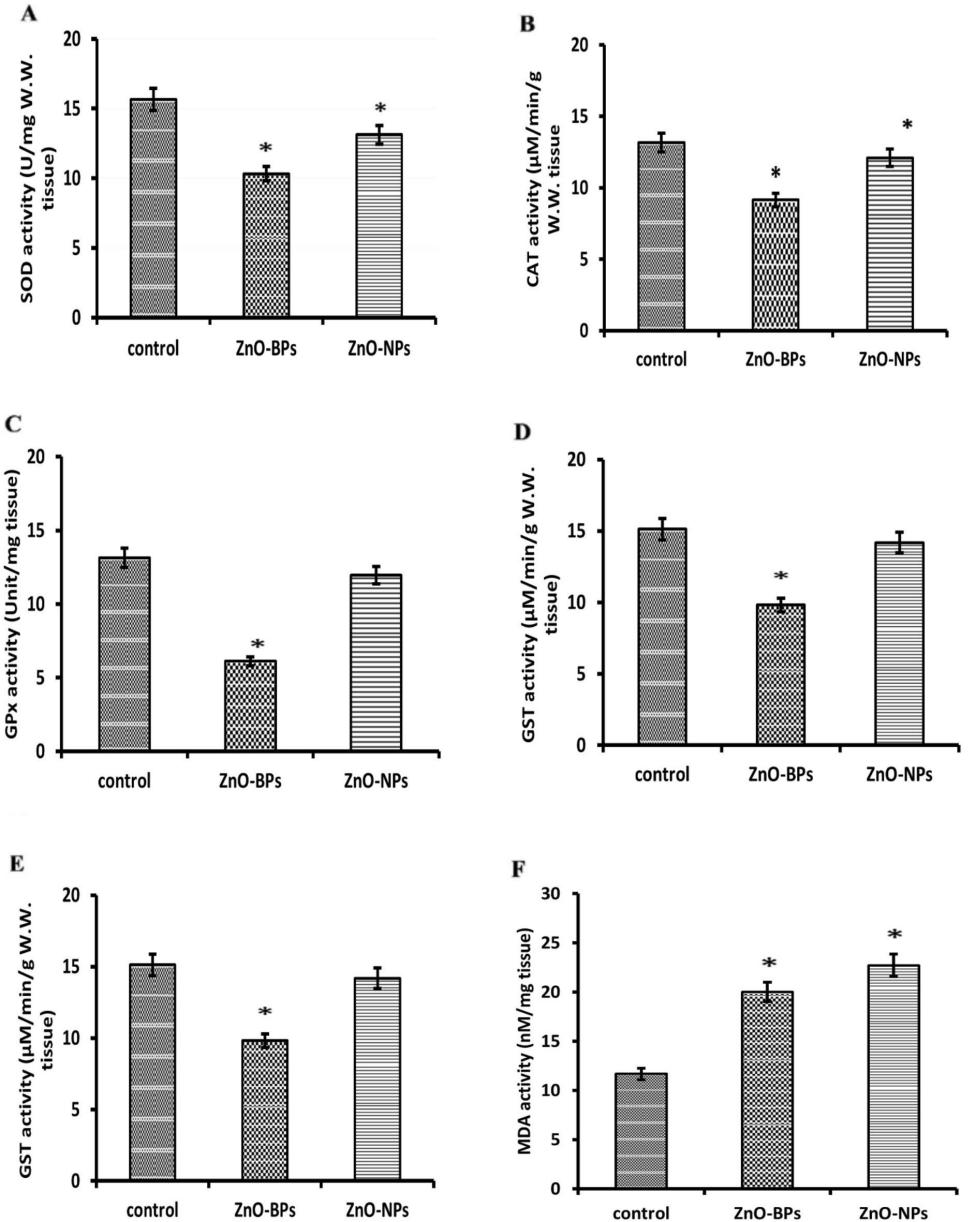
SOD, CAT, GPx, GST activities, GSH content, and MDA level in control and treated veliger larvae with 10 µM ZnO-BPs and 0.4 µM ZnO-NPs. *p < 0.05 versus control. (**A**) Superoxide dismutase (SOD). (**B**) Catalase (CAT). (**C**) Glutathione peroxidase (GPx). (**D**) Glutathione S-transferase (GST). (**E**) Reduced glutathione (GSH). (**F**) Malondialdehyde (MDA).

SOD, CAT, GPx, GST activities, GSH content, and MDA level in control and treated veliger larvae were investigated and illustrated in Table 6 and Fig. 9. The data revealed a significant difference in all studied oxidative biomarkers, except GPx and GST, after exposure to ZnO-NPs. The exposure of *H. duryi* veligers to ZnO-BPs and ZnO-NPs showed a significant decrease in the activities of both SOD and CAT compared to the control groups (Fig. 9A,B). Regarding the GPx and GST activities, the results showed a significant decrease after exposure of larvae to 10 µM ZnO-BPs. However, ZnO-NPs cause a decrease but are insignificant in both enzymes (Fig. 9C,D), indicating that ZnO-BPs had higher oxidative stress than ZnO-NPs.

**Table 6:** Comparison between oxidative stress parameters in control and treated egg masses with ZnO (both bulk and nano forms).

Oxidative stress parameter	Control	ZnO-BPs	ZnO-NPs
SOD	15.65 ± 0.32	10.31 ± 0.63*	13.11 ± 0.93*
CAT	13.2 ± 0.59	9.14 ± 0.39*	12.1 ± 0.44*
GPx	13.13 ± 0.66	6.11 ± 1.13*	12.1 ± 2.27
GST	15.12 ± 1.57	9.81 ± 0.88*	14.19 ± 0.29
GSH	24.24 ± 0.91	10.13 ± 0.24*	4.99 ± 0.43*
MDA	11.68 ± 0.79	20.02 ± 1.13*	22.72 ± 0.65*

*Significant (*p* < 0.05) versus control.

GSH activity showed a significant decrease in both treated groups in comparison with the control group (Fig. 9E). These data revealed the more significant oxidative damage of ZnO-NPs than ZnO-BPs.

As shown in Fig. 9F, both ZnO forms significantly increase the MDA levels in veligers. This elevation in MDA level indicates increased LPO, which may explain the reduction in the total lipids level. MDA levels in the exposed veligers to ZnO-NPs and BPs were 22.72 ± 0.65 and 20.02 ± 1.13, respectively, revealing that ZnO-NPs had more antioxidant stress than ZnO-BP, Table 6.

#### Effect of both ZnO forms on the organic content of veliger larvae

Data analysis of control and treated veliger exposed to 10 µM ZnO-BPs and 0.4 µM ZnO-NPs showed a significant difference in all studied organic substances.

**Figure 10:**
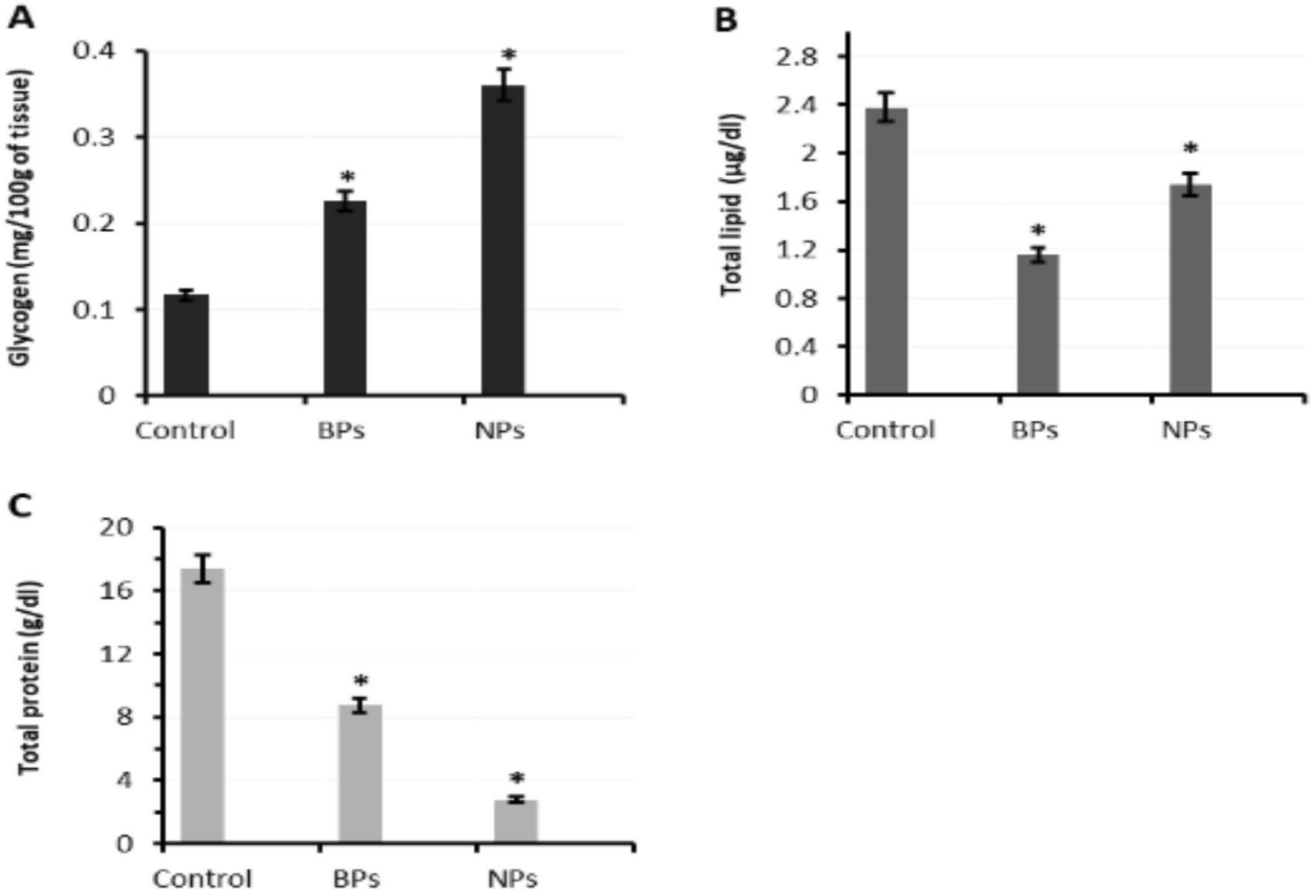
Biochemical effects of both ZnO forms (BPs = Bulk and NPs = Nano) on (**A**). glycogen level (**B**). total lipid (**C**). total protein of veliger larva. *p < 0.05 versus control.

As shown in Table 7, exposure of egg masses to 10 µM ZnO-BPs and 0.4 µM ZnO-NPs resulted in a significant increase in glycogen level. The increase ratio reached 93.16% and 207.69%, respectively, indicating that ZnO-NPs had more toxic effects than ZnO-BPs (Fig. 10A). The exposed egg masses to ZnO-BPs and ZnO-NPs showed a significant decrease in total lipids, compared to control groups, with a ratio of decrease reaching 51.26% and 26.89%, respectively (Fig. 10B). The obtained data also showed a significant decrease in the total protein of ZnO-BPs and NPs treated groups compared with their matched control group. The decrease ratio reached 49.54% and 83.99%, respectively, indicating that ZnO-NPs had higher effects than ZnO-BPs (Fig. 10C).

**Table 7:** The effect of ZnO-BPs (10 µM) and ZnO-NPs (0.4 µM) on biochemical parameters of egg masses of *H. duryi* containing veliger larvae.

Group	Glycogen (mg/ 100 g of tissue)	Percentage change	Total lipid (μg/dl)	Percentage change	Total protein (g/dl)	Percentage change
Control	0.117 ± 0.01		2.38 ± 0.26		17.36 ± 1.9	
ZnO-BPs treated veliger	0.226 ± 0.023*	93.16% ↑	1.16 ± 0.13*	51.26 ↓	8.76 ± 1.9*	49.54% ↓
ZnO-NPs treated veliger	0.36 ± 0.036*	207.69% ↑	1.74 ± 0.14*	26.89 ↓	2.78 ± 1.4*	83.99% ↓

*Significant (*p* < 0.05) versus control.

### Effect of both ZnO forms (bulk and nano) on the veliger protein profile

**Figure 11:**
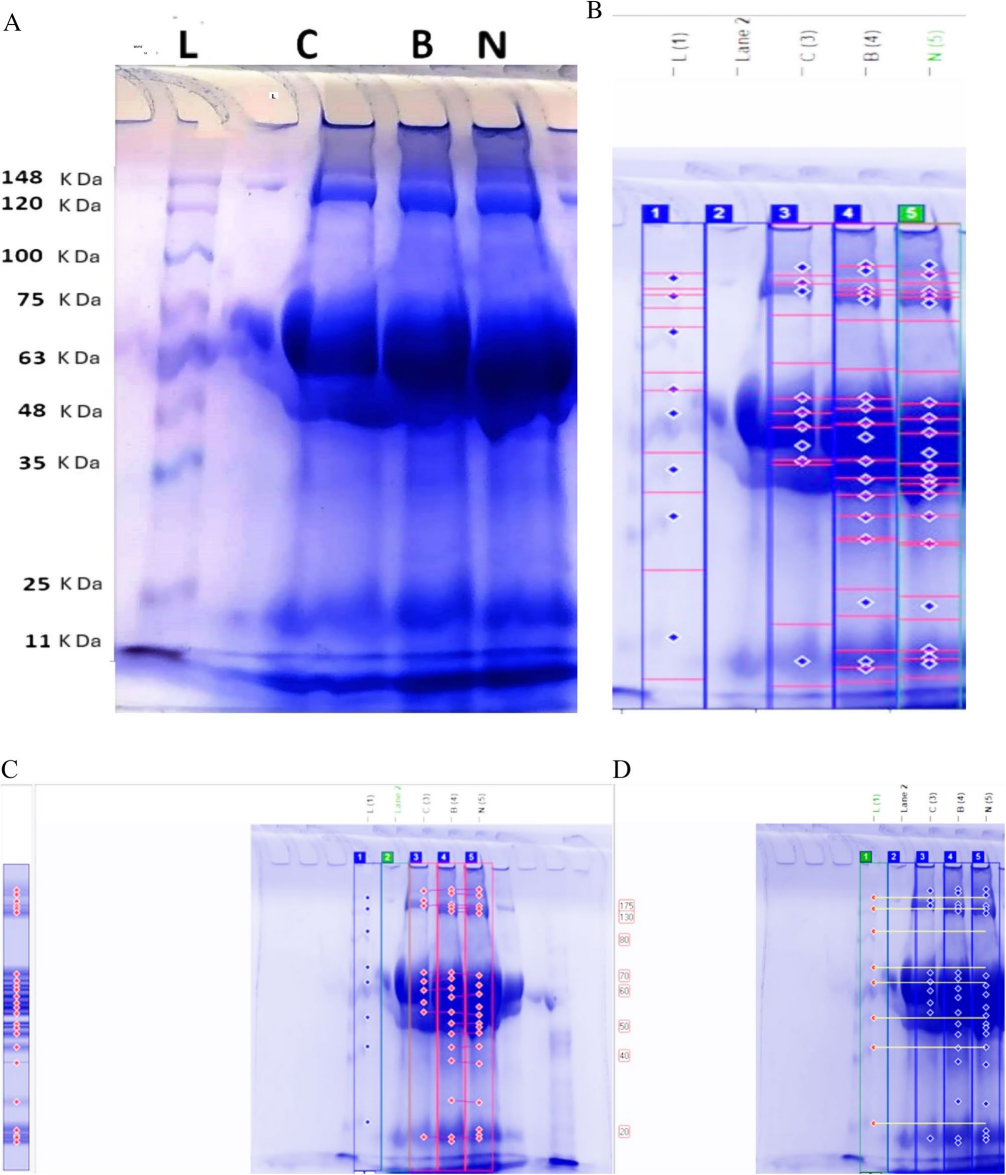
SDS-PAGE profile of total protein of veliger larva of *H. duryi* stained with Coomassie brilliant blue (**A**), bands computerized detection for sample (**B**), computerized loci detection for samples (**C**), molecular weight calculation for samples (**D**). C = control veliger, B = 10 µM ZnO-BPs exposed veliger larvae, N = 0.4 µM ZnO-NPs exposed veliger larvae, and L = (Ladder).

**Figure 12:**
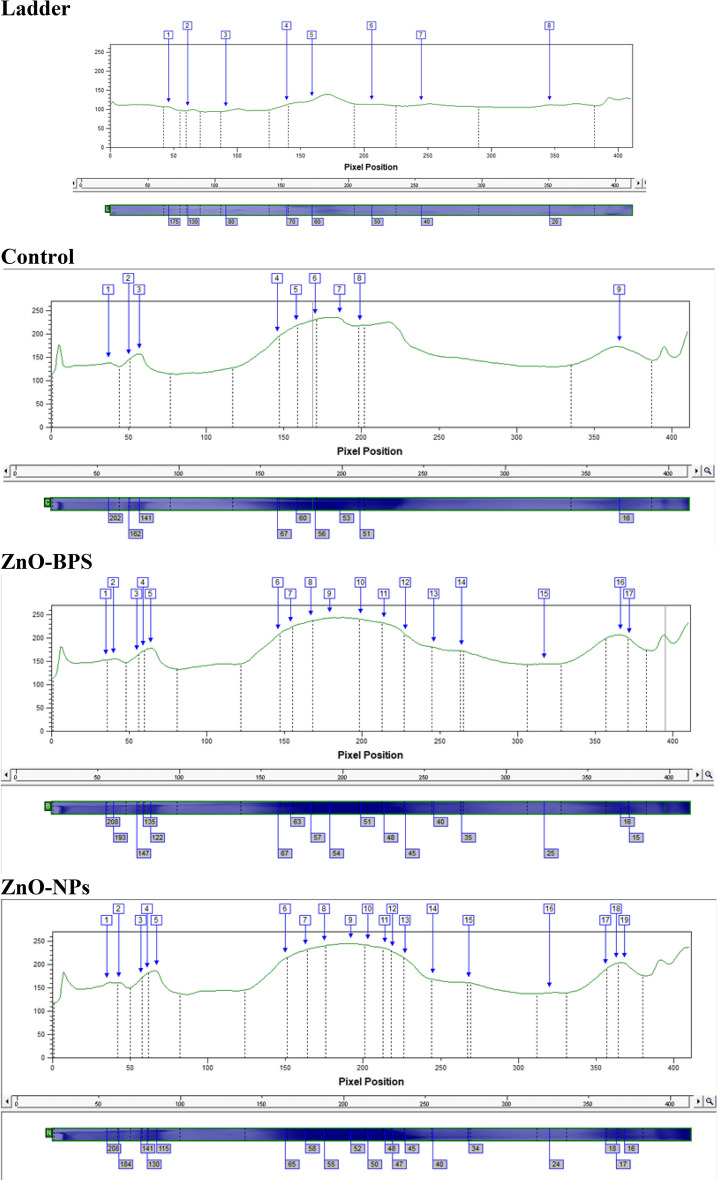
Dendogram for protein pattern of ladder and samples.

**Figure 13:**
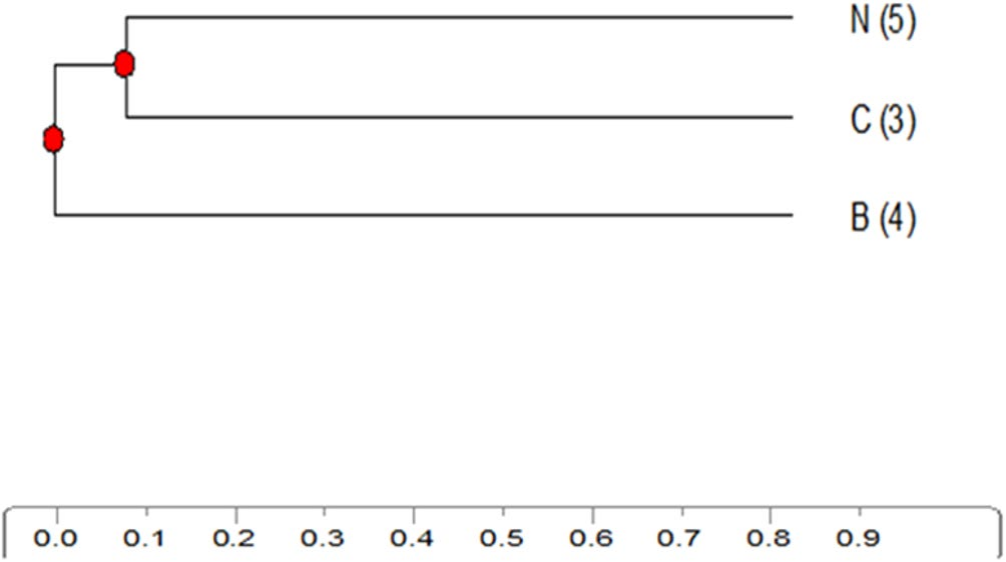
phyllogenetic tree for control and treated samples according to protein patterns.

The analysis of the gel-electrophoretic protein profile of the control and treated veliger larvae showed considerable variations. The results of gel separation of protein and their scanning are presented in Table 8 and Figs. 11, 12 and 13 (Supplementary files 1 and 2).

**Table 8:** SDS-PAGE of proteins of control (C) and 10 µM ZnO-BPs (B) and 0.4 µM ZnO-NPs (N) exposed veliger larvae.

Band No	Volume	Peak Height	Area	Band %	MW	Rf
C						
1	209,661.00	138.56	1548.00	16.51	*202.000*	0.090
2	34,391.00	145.06	252.00	2.71	162.339	0.122
3	125,557.00	157.31	936.00	9.88	141.095	0.139
4	166,561.00	192.83	1080.00	13.11	66.942	0.356
5	89,595.00	217.22	432.00	7.05	60.487	0.385
6	97,201.00	230.53	432.00	7.65	55.871	0.415
7	222,970.00	234.36	972.00	17.55	52.688	0.454
8	31,464.00	218.22	144.00	2.48	51.044	0.485
9	292,866.00	173.08	1872.00	23.06	*16.040*	0.893
B						
1	187,470.00	153.25	1260.00	10.31	*208.000*	0.085
2	66,110.00	155.03	432.00	3.64	*193.000*	0.098
3	44,351.00	162.69	288.00	2.44	146.975	0.134
4	24,312.00	171.53	144.00	1.34	135.424	0.144
5	117,769.00	178.25	756.00	6.48	122.386	0.156
6	155,992.00	207.31	900.00	8.58	66.942	0.356
7	62,448.00	222.44	288.00	3.43	62.588	0.376
8	107,972.00	236.31	468.00	5.94	56.794	0.407
9	261,535.00	243.22	1080.00	14.38	53.796	0.437
10	128,014.00	240.61	540.00	7.04	51.044	0.485
11	113,394.00	231.89	504.00	6.24	48.405	0.522
12	124,512.00	207.44	648.00	6.85	44.823	0.556
13	113,796.00	180.39	648.00	6.26	39.725	0.600
14	12,446.00	172.75	72.00	0.68	35.143	0.644
15	114,505.00	144.64	792.00	6.30	24.701	0.773
16	103,153.00	206.86	504.00	5.67	*16.040*	0.893
17	80,557.00	198.64	432.00	4.43	*14.851*	0.907
N						
1	224,288.00	160.19	1476.00	11.56	*208.000*	0.085
2	45,096.00	160.83	288.00	2.32	*184.000*	0.105
3	45,811.00	169.92	288.00	2.36	141.095	0.139
4	25,496.00	180.92	144.00	1.31	130.000	0.149
5	117,081.00	186.64	720.00	6.03	115.396	0.163
6	170,753.00	213.08	972.00	8.80	64.798	0.366
7	104,733.00	230.86	468.00	5.40	58.251	0.398
8	102,128.00	239.81	432.00	5.26	54.606	0.427
9	219,311.00	244.97	900.00	11.30	51.918	0.468
10	103,242.00	241.42	432.00	5.32	50.482	0.495
11	4 2 065.00	234.83	180.00	2.17	48.405	0.522
12	64,338.00	227.69	288.00	3.32	47.216	0.534
13	123,706.00	212.31	648.00	6.37	45.100	0.554
14	135,795.00	168.67	828.00	7.00	40.000	0.598
15	11,580.00	160.58	72.00	0.60	34.213	0.654
16	95,325.00	139.67	684.00	4.91	24.198	0.780
17	148,555.00	189.75	936.00	7.66	*18.020*	0.868
18	49,896.00	202.36	252.00	2.57	*16.634*	0.885
19	111,341.00	203.33	576.00	5.74	*15.644*	0.898

Significant values are in (italics).

The results indicated that the protein pattern of polypeptides contained 9, 17 and 19 bands for control, ZnO-BPs, and ZnO-NPs exposed egg masses, respectively. The molecular weights ranged from 14 to 208 kDa. It was noticed that three shared bands between control and ZnO-BPs exposed egg masses. In comparison, the shared bands between control and ZnO-NPs exposed egg masses decreased to one band. The results showed that 6 proteins disappeared from the treated ZnO-BPs group and 8 appeared. On the other hand, 8 proteins disappeared from the treated ZnO-NPs group and 10 appeared.

The cluster analysis (phylogenetic tree) applied on the obtained data (Fig. 13) revealed the degree of similarity among the control and treated groups. The control and ZnO-BPs share a closer branch due to higher similarity while ZnO-NPs branches out further due to lower similarity.

## Discussion

Previous studies have increasingly recognized the potential embryotoxicity of ZnO-NPs, particularly in aquatic organisms. Gastropods, including freshwater snails like *H. duryi*, are sensitive indicators of environmental stressors, including nanoparticle contamination. Research on the toxicity of ZnO-NPs to gastropods has shown adverse effects on growth, reproduction, and immune function, highlighting their potential ecological impact^59^. ZnO-NPs can readily penetrate biological barriers and interact with cellular components, leading to adverse effects on embryonic development. The mechanism underlying ZnO NP-induced embryotoxicity involves complex cellular and molecular processes. Oxidative stress is a prominent mechanism, wherein ZnO-NPs generate reactive oxygen species (ROS), causing cellular damage and disrupting essential biochemical pathways. Additionally, ZnO NPs may induce genotoxicity, inflammation, and apoptosis, further exacerbating developmental abnormalities in exposed embryos^60^.

Several studies have suggested that the release of Zn^2+^ ions mainly cause the toxicity of ZnO (bulk and nano) and depends on release rates in time. Solubility is a fundamental parameter as it may result in the delivery of extremely harmful ions^61,62^. Others suggest that the intrinsic properties of ZnO are responsible for its toxicity. The present results, due to the limited solubility of bulk and nano-zinc oxides, suggest that both factors are responsible, as also previously mentioned^7,63,64^.Moreover, the toxicity of NPs is affected by various factors such as shape, size, and chemical composition^65,66^. The toxicity of ZnO-NPs increases with increasing concentration or decreasing particle size. Thus, higher toxicity was due to higher levels of ZnO-NPs induced intracellular oxidative stress^6^. The lethality of snails' embryos was due to the easy penetration of the smallest ZnO-NPs of chemicals inside the eggs^67^. Our results agree with them as they confirmed that ZnO-NPs are more toxic to the embryo than ZnO-BPs because of their particle size, less than 31 nm.

The induction of malformations in developing embryos is a common effect observed after snail embryo exposure to metal NPs^27,30^. In the present work, the major alteration observed after exposure to both ZnO forms was hydropic malformation (embryo partly or totally swollen). Exposure to both ZnO forms probably leads to the uptake and retention of water in the embryo and the gradual development of hydropic changes. The increased frequency of hydropic malformation was observed after exposure to both ZnO forms compared to the control group.

Hydropic embryos rarely hatched or frequently died after hatching^67^. Furthermore, the hydropic malformations were associated with developmental retardation of snail embryos^68^, indicating potential neurotoxic effects of both ZnO forms on *H. duryi* embryos. A similar pattern of developmental inhibition induced by Zn ions was observed in other aquatic organisms, such as zebrafish^69–71^.

The teratogenic effect of ZnO at varying stages and concentrations reveals that gross congenital malformations are formed in embryos, especially at the veliger stage. This stage can be considered the specific stage at which the embryos are much more sensitive to both ZnO forms. These findings agree with Gomot^72^, who found that the pulmonate snail veliger stage was considered the most sensitive stage in toxicity studies. Similarly, silver NPs inhibited the development of the gastrula and veliger stages of the *B. glabrata* after 144 h of exposure^47^.

More recently, Mehraj and Pandit^73^ found that exposure to copper NPs (Cu-NPs) impairs the veliger stage in the snail *Indoplanorbis exustus*. Also, after *Lymnaea luteola* was exposed to Cu, the abnormality was observed at the veliger stage^74^ and more prominent at the hippo stage^75^. The researchers concluded that embryos at the hippo stage were more susceptible to nanotoxicity when compared to the blastulae, indicating the higher susceptibility of larval stages to NPs than other embryonic stages. Thus, both ZnO forms' toxicity may depend on the variation of developmental stage, as mentioned for silver NPs^47^. Additionally, in other bivalves, a similar developmental inhibition was induced by bulk Ag and its NPs^76,77^. However, previous results confirmed that in mollusks, the veliger stage can represent a sensitive target for certain types of metals (bulk or nano).

The question raised in the present study is how veliger larvae are more sensitive to ZnO than the early developmental stages. The egg cell of *H. duryi* benefits from two levels of protection, the double gelatinous outer layer surrounding it and the envelope (vitelline membrane) in which the embryo can survive. So, the present study supposed that ZnO particles accumulate and contact the cell surface in one way. Adherence between chemical particles, and the cell wall is sufficient to cause toxicity by generating extracellular ROS that may damage the cell membrane^78^.

However, as the development proceeds, the protective layers around the eggs become weak and permeable to prepare the embryo for hatching. In this case, the chemical particles can penetrate through the cellular barrier and accumulate in the organs and tissues of the embryo, causing more pronounced pathomorphological alterations. This explanation agrees with other observations^71,79,80^. Zn is bound to the egg’s shell during the early embryonic stages and is not in direct contact with the embryo. Then, the shell becomes permeable after organogenesis, and the metals can exert their toxicity on the embryo.

It is imperative to evaluate the toxicological mechanisms of both forms of ZnO (bulk and nano) on the specific stage, veliger larva. Oxidative stress was the main toxic effect induced by NMs in gastropods^16^. So, it is a convenient parameter to measure toxicity because cells respond to it with many defensive responses that are easily measured as increasing ROS production, altered enzymatic activities, and genetic expression^81^. Furthermore, NMs can also induce protein carboxylation and reduce the total protein content^37^.

Malondialdehyde (MDA) is the end product produced metabolically by lipid peroxidation (LPO)^82^. It is considered an important parameter that provides a relative way to evaluate the level of oxidative stress caused by metal oxides and their NPs in snails^6^. The current study indicates damage caused in some veliger cells, as ZnO generally caused significant elevation of MDA in the egg masses. So, these results agree with the previous findings^6,7,36^ revealed that ZnO-NPs elevated MDA and caused damage in some tissues of some freshwater snails. Also, the present results coincide with other results^83^ in which lipid peroxidation levels increased in mussel tissues due to copper metal exposure. The correlation between ROS and MDA suggests that increased generation of ROS by ZnO-NPs causes oxidative damage in embryos, followed by teratogenesis^84^.

Superoxide dismutase (SOD) is usually used as the first biomarker to indicate oxidative stress^85^. The activity of SOD would increase to eliminate the raised ROS and to keep its production at a steady-state concentration in cells^86^. In the present study, ZnO (BPs and NPs) treated groups showed lower SOD activities than the control group, and this result agrees with another study on the effect of ZnO-NPs on freshwater snail *B. alexandrina*^7^. The inhibition indicates an oxidative stress condition, which may arise due to an imbalance in ROS formation and cells' antioxidant defense system^87^. SOD aids in the breakdown of, a free radical, superoxide (O^-2^) by converting it to H_2_O_2_^88^.

Concerning glutathione, reduced glutathione (GSH) is one of the most important factors protecting from oxidative attacks by ROS because GSH acts as a reducing agent and free-radical trapper and is known to be a cofactor substrate for many enzymes^89^. The decline of GSH in the present study confirmed the previous findings^6,90^, which suggested that the decrease in GSH content in the digestive gland of mollusks appears to be a common response to metal exposure, which can partly be justified by the high affinity of Zn metal for the GSH molecule. Also, GSH levels decreased in the digestive gland and kidney in zinc-treated *Achatina fulica* snails^91^.

Consequently, the present investigation showed a significant decrease in GPx and GST in ZnO-treated snails compared with the control group. The inhibition of both enzyme activities may be due to the direct action of metal on the enzyme or overproduction of ROS and thus depletion of their substrate GSH. The inhibition of GPx and GST levels in the present study comes in agreement with other studies^6,7^.

CAT decomposes H_2_O_2_ into water and oxygen to prevent oxidative stress and maintain cell homeostasis^92^. Thus, the SOD-CAT system provides the first defense against oxygen toxicity^84^. In the present study, a significant decrease in CAT was observed, indicating the adverse effect of ZnO exposure on CAT activity. It could be due to the enhancement of the peroxidation end product (MDA), which inhibits protein synthesis and the activities of certain enzymes^93^. Variable CAT responses to metal exposure have been found, with some snails exhibiting depressed activity, others exhibiting increased activity, and others showing no CAT response at all, as previously mentioned^6,36,37^. The continual exposure to toxic metals and the flux of ROS can induce severe disturbance of CAT activity in tissues by binding these metal ions to –SH groups of the enzyme^6^. Accordingly, this leads to the overproduction of H_2_O_2_, CAT activity alteration, oxidative damage, and teratogenesis.

Total lipid reduction in the current snail embryos exposed to ZnO-NPs, agrees with previous findings in other aquatic organisms^94,95^. This inhibition was possibly caused by toxicant stress, which increased metabolic rate, thus decreasing metabolic reserves. Due to the reduced energy content, the reduced total lipid content inhibited the development of the snail embryo^96^. Contradictory to these results, other authors found a considerable increase in total lipids in snail hemolymph and tissues after ZnO-NPs exposure^7,97^.

The current study reveals a reduction of total proteins after treatment with ZnO-NPs, which agrees with other studies^7,37^ which attributed this decline to the fact that NPs can cause protein carboxylation. Conversely, this decrease could result from the breakdown of protein into amino acids, nitrogen, and other elementary molecules^98^. However, the denaturation of proteins in cells results in the disruption of cell activity and possible death^98^. Besides their high sensitivity to metal poisoning, veliger proteins play critical functions such as shell formation, body torsion, nervous system development, muscular system differentiation, metamorphosis, and immune-related activities^99^. Consequently, ZnO indirectly affected these functions as it caused a total protein decrease, leading to embryo inhibition.

ZnO inhibited protein synthesis. Accordingly, Sodium dodecyl sulfate–polyacrylamide gel electrophoresis (SDS-PAGE) was used to assess the effects of ZnO (bulk and nano) on protein synthesis^37^. ZnO-NPs qualitatively influenced the protein patterns of eggs containing veligers. ZnO-BP caused the disappearance of 6 proteins and appearance of 8 proteins. On the other hand, ZnO-NPs caused disappearance of 8 proteins and appearance of 10 protein. These changes indicated that the tested substance caused intensive molluscicidal effects that induced fractionation of the native protein^100^. The similarity between the control and two ZnO-tested groups revealed that the control was more similar to the ZnO-BPs than the ZnO-NPs, reflecting the higher effectiveness of the nano form than the bulk (Supplementary files 1 and 2).

## Conclusion

This study compares the toxic effects of bulk zinc oxide (ZnO-BPs) and its NPs (ZnO-NPs) on *H. duryi* embryos, revealing ZnO-NPs induce more severe developmental defects. Mechanistically, lipid peroxidation and oxidative stress plays a key role, altering biomarkers and organic substances crucial for embryonic development*. H. duryi* embryos prove effective for assessing ZnO-NP toxicity in aquatic environments, highlighting the importance of nanoparticle properties in toxicity evaluations. These findings deepen our understanding of ZnO nanoparticle toxicity in aquatic organisms and advocate for further research to mitigate environmental impacts.

### Supplementary Information

Supplementary Information 1.

Supplementary Information 2.

## Data Availability

Data will be made available by the corresponding author on request.
