# Supplementary figures and images for: Evaluation of the teratogenic potency of bulk zinc oxide and its nanoparticles on embryos of the freshwater snail, Helisoma duryi
Source: Sci Rep. 2024 Jul 10;14:15888. doi: 10.1038/s41598-024-66008-x (PMC11237064; doi:10.1038/s41598-024-66008-x)

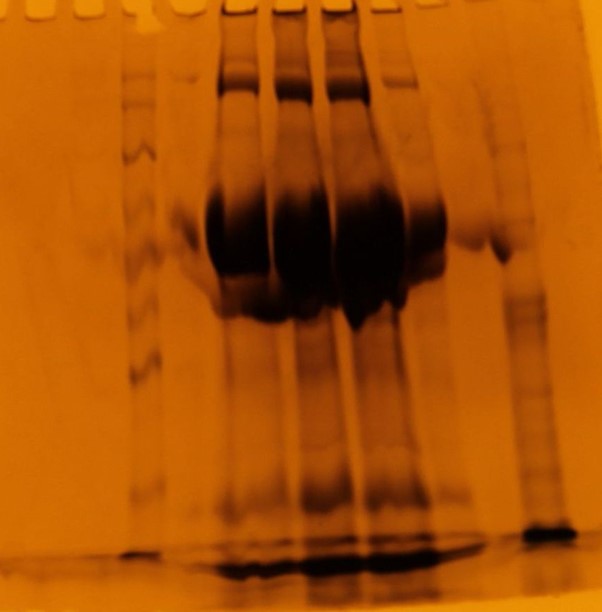

Supplement: Supplementary file 1 — Supplementary Information 1. [file 41598_2024_66008_MOESM1_ESM.jpg]

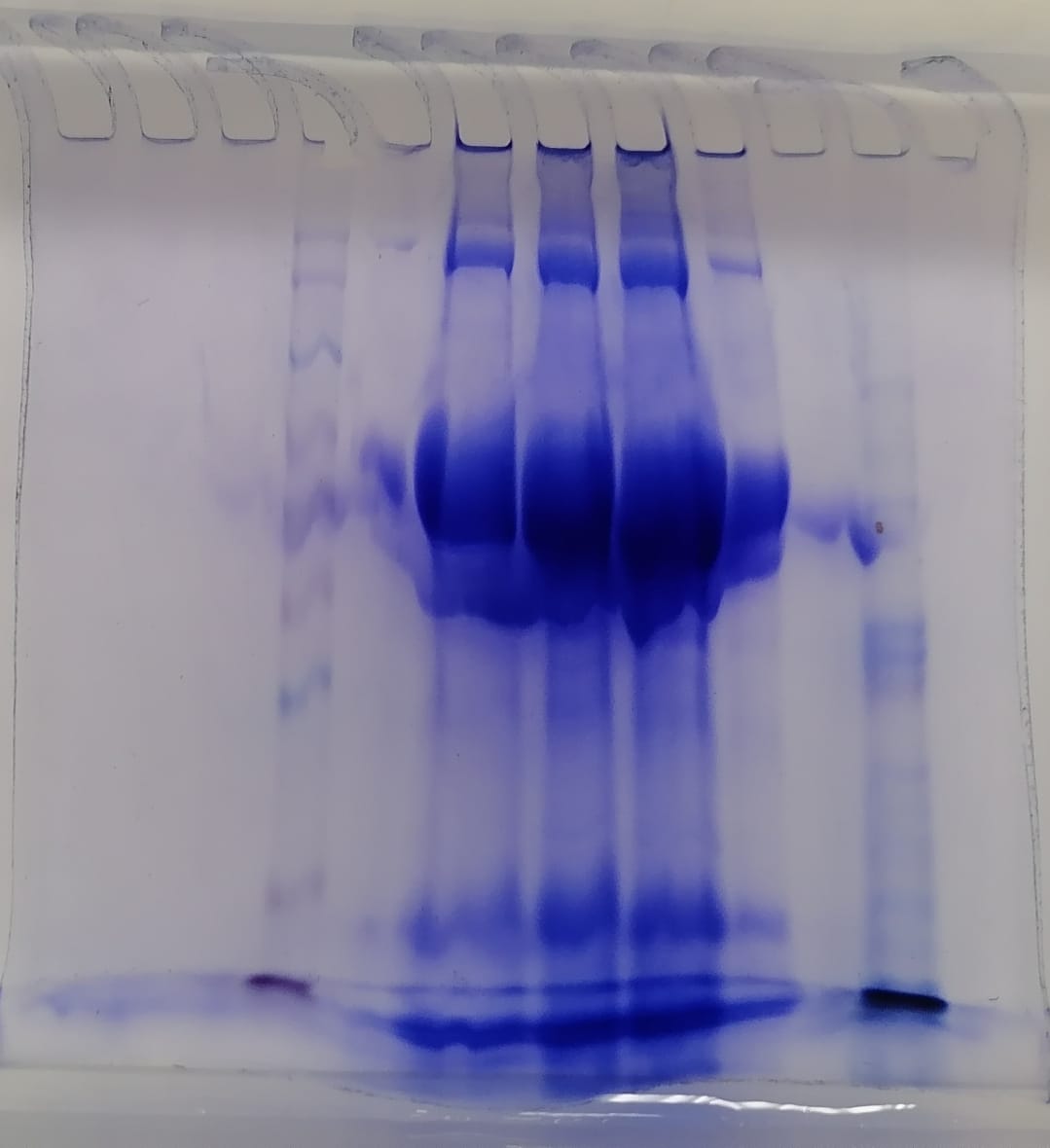

Supplement: Supplementary file 2 — Supplementary Information 2. [file 41598_2024_66008_MOESM2_ESM.jpg]
